# A Transparent, Simple, Audit-Oriented Python-Based Binary Allocation Prototype for Clinical Research

**DOI:** 10.7759/cureus.108209

**Published:** 2026-05-03

**Authors:** Kevin T Malone

**Affiliations:** 1 Emergency Medicine, Baylor Scott & White Medical Center - Temple, Temple, USA

**Keywords:** audit, random, randomization, research, transparent

## Abstract

Background

Clinical randomization requires more than approximate 1:1 allocation. It also requires sequence generation that is difficult to subvert, allocation concealment during enrollment, and an audit trail that can withstand retrospective review.

Objective

The primary objective of this proof-of-concept technical and methodological evaluation was to assess the inspectability and audit-oriented design of a lightweight Python-based two-arm allocation prototype. Secondary objectives were to characterize its short-run demonstration behavior and compare its transparency, traceability, and operational limitations with common clinical randomization workflows.

Methods

We performed a static review of the supplied Python source file and a retrospective review of the supplied allocation log. Log analysis included descriptive arm counts, exact binomial testing for 1:1 balance, an exploratory runs test, and lag-1 autocorrelation. The implementation was interpreted in light of the clinical-trial randomization and pseudorandom-number-generator literature.

Results

The current source code implements a lightweight two-arm allocation randomization prototype with features intended to support auditability and tamper-evident traceability. Each allocation advances a xorshift-inspired 64-bit generator, maps the resulting integer to arm 1 or 2, and records the underlying output and assignment arithmetic in a human-readable log. An example test log was run and contained 2,000 allocations, with 502 versus 498 assignments in the first 1,000 events and 1008 versus 992 assignments overall. The current source code also implements session seed capture and a chained SHA-256 digest intended to strengthen sequential traceability and show evidence of tampering.

Conclusions

The prototype’s principal improvement over many ad hoc local workflows is operational transparency rather than proven statistical superiority. Its strongest contribution is a short, inspectable code path and an audit-oriented logging structure. Additional hardening would be required before use in concealment-sensitive or regulated trial settings.

## Introduction

Randomization reduces selection bias in clinical research, but good randomization practice involves more than achieving an approximately equal number of participants in each study arm. Sequence generation and allocation concealment are related but distinct requirements, and both are essential if a trial is to resist manipulation during enrollment [1-3].

Clinical investigators may use simple randomization, block randomization, stratified designs, or covariate-adaptive methods such as minimization. These approaches differ in how they balance unpredictability, group balance, covariate balance, and operational complexity [4-9]. In practice, however, many research teams still rely on ad hoc spreadsheets, desktop tools, or locally written scripts because these options are inexpensive, accessible, and easy to deploy. Such tools may be useful, particularly for pilot studies or single-center projects, but they vary considerably in transparency, reproducibility, and audit support [10-13]. The Malone Randomizer is a binary allocation prototype designed to emphasize transparency. It is written in Python, uses a minimal desktop interface, returns a treatment code of 1 or 2 on demand, and writes a text log showing the integer output used to generate the assignment. The central scientific question is therefore not whether a short empirical sequence can prove randomness, because finite statistical testing cannot establish that conclusively [14,15]. Rather, the more relevant question is whether the tool improves clinical randomization practice by making the allocation process more inspectable and easier to review. This manuscript has four aims: first, to describe the program; second, to explain how the code works at both high and low technical levels; third, to characterize what the supplied log does and does not demonstrate; and fourth, to compare the prototype with more established approaches to clinical randomization.

Study objectives

The primary objective of this study was to evaluate whether the Malone Randomizer provides an inspectable, audit-oriented implementation of simple binary allocation. Secondary objectives were to describe the prototype’s software architecture, characterize the supplied 2,000-event demonstration log, identify deployment-relevant limitations, and compare the prototype with common randomization workflows.

System overview and design rationale

At a functional level, the Malone Randomizer is a simple two-arm allocation tool. A user launches the application, clicks the Randomize button, receives an assignment coded as 1 or 2, and is shown a short text record displaying the raw generator output and the arithmetic used to convert that output into the assignment.

The current source code relies only on Python standard-library modules: tkinter for the graphical interface, datetime for timestamps, hashlib for SHA-256 hashing, and os for file handling. This low-dependency design is a practical advantage because the tool can run on a standard Python installation without external scientific packages or database infrastructure.

Its novelty is operational rather than theoretical. The program does not introduce a new randomization method such as blocked, stratified, or minimization-based allocation [6-9], nor does it establish a new class of validated pseudorandom number generator. Instead, its main contribution is a transparent implementation in which the path from generator state to treatment assignment is short, inspectable, and explicitly documented for review.

The central aim of this prototype is inspectability. Reproducing the code in full allows readers to review the exact implementation of seed generation, pseudorandom number generation, assignment mapping, chained logging, and interface behavior in a single place. The full source code and test data are publicly available in the Malone Randomizer GitHub repository, and the Appendix summarizes the core program components and logged fields. The central algorithmic object in the program is a class named RawXORShiftPRNG, best described as a xorshift-inspired 64-bit pseudorandom number generator. The shift triplet (12, 25, 27) and multiplier 0x2545F4914F6CDD1D are recognizable from the xorshift* literature [16,17]. The source code uses four Python standard-library imports: tkinter for the graphical user interface, datetime for timestamp generation, hashlib for chained SHA-256 hashing of log entries, and os for basic file-handling operations. These modules support the interface and logging architecture, whereas the allocation logic itself is implemented directly in the program source rather than through an external randomization library.

In canonical xorshift* formulations, the multiplicative scrambling step is applied to the returned output, while the underlying linear state is updated separately [17]. In the present implementation, by contrast, the multiplied value is written back into the internal state itself. This does not make the generator unusable, but it does mean that the formal period and empirical test-performance results reported for canonical xorshift* generators cannot be assumed to apply unchanged to this implementation.

The seed is derived from the current timestamp, converted to a decimal string, and mixed character by character into a 64-bit integer. This approach is lightweight and reproducible, but it is not cryptographically strong. Although cryptographic strength is not the primary aim of the present proof-of-concept, approximate knowledge of launch time could materially narrow the seed space. In a clinical research setting, that matters because predictability and allocation concealment are operational as well as mathematical concerns [2,3].

Allocation is determined by the parity of the generated integer: if val % 2 equals 0, the program assigns arm 1; otherwise, it assigns arm 2. This rule is attractive because it is simple, transparent, and deterministic. It is also a limitation, because only a single bit of each 64-bit output determines the final assignment. In analyses of xorshift* generators, the least significant output bit is recognized as a weak point because it is not materially improved by the multiplicative scrambling step [17]. Because the multiplier used here is odd, parity is preserved at line 24, so the treatment assignment effectively depends on that least significant bit.

The logging architecture is one of the strongest features of the present prototype. Each allocation is recorded as a text entry containing a timestamp, the raw integer output, and the explicit arithmetic used to map that output to arm 1 or arm 2. The current source code also computes a chained SHA-256 digest that incorporates the previous entry’s hash, thereby linking each event to the one before it. Audit trails are central to traceability in health information systems [18,19], and chained digests are consistent with established approaches to secure logging and linked timestamping [20,21].

At the same time, the audit implementation remains local and user-resettable. The clear_log() function overwrites the file, resets the counter, and clears the chain state. Accordingly, a local hash chain without external notarization, write-once storage, or trusted timestamping may improve detection of later edits, but it does not by itself create immutability [20,21].

The user interface is deliberately minimal. It includes a button to generate an allocation, a button to clear the log, and a text box displaying the most recent entry. This simplicity is useful for demonstration and lightweight deployment, but it also means that the current prototype lacks patient identifiers, user authentication, role separation, a central allocation service, and an irreversible commit step before assignment is revealed. In real clinical trial operations, these omissions are not merely cosmetic; they bear directly on allocation integrity [2,3].

## Materials and methods

This study was designed as a proof-of-concept technical and methodological evaluation of an inspectable binary randomization prototype, rather than as a clinical trial, regulatory validation study, or assessment of patient outcomes. The analysis was based on two study artifacts: a Python source file (Randomizer.py) and a retrospective demonstration log (randomizer_log.txt). The source file contained 107 lines. The demonstration log contained 2,000 sequential allocation events recorded between July 22, 2025, 00:15:07 and 00:24:05; these analyses are illustrative and not intended to validate the generator. The analytic structure followed the stated objectives: static code review addressed the primary objective of inspectability and audit-oriented design; retrospective log analysis addressed short-run demonstration behavior; and literature-based comparison addressed deployment limitations and the prototype’s position relative to established randomization approaches.

The code review focused on six domains: seed generation, internal state update, output-to-assignment mapping, audit-log construction, user-interface behavior, and implications for clinical research deployment. The implementation was then interpreted in light of the clinical-trial randomization and pseudorandom-number-generator literature [1-9,14-17].

The log review focused on descriptive sequence behavior. We counted assignments to arms 1 and 2 across both the first 1,000 events and the full 2,000-event sequence, tested the observed arm-1 counts against the null expectation of 0.5 using exact binomial tests, performed an exploratory Wald-Wolfowitz runs test, and estimated lag-1 autocorrelation. These statistical analyses were used descriptively and illustratively to characterize the supplied demonstration log, not as confirmatory validation of the generator or proof of randomness.

The current source file writes session headers, the initial seed, a Used Bit field, and a Hash Chain field, whereas the supplied demonstration log contained only the result, raw integer output, and arithmetic trace. The two artifacts therefore appear consistent with the same underlying allocation logic, but not perfectly matched in audit-log format. That discrepancy is itself important, because alignment between executable code, logging schema, and demonstration output is part of any credible audit framework. Exact computational reproducibility of the complete audit trail is therefore limited. Although the current source file describes the seed-generation approach, PRNG state-transition logic, assignment mapping, and chained logging structure, the 2,000-event demonstration log should be interpreted as an illustrative retrospective allocation sequence rather than a complete hash-chained audit-log export from the current source-code version shown in the manuscript.

## Results

The supplied log contained 2,000 sequential allocations. Arm 1 was assigned 1,008 times (50.4%) and arm 2 was assigned 992 times (49.6%). In the first 1,000 allocations, the split was 502 versus 498.

Exact binomial testing showed no detectable departure from a 1:1 null ratio in either window (P=0.92 for the first 1,000 allocations and P=0.74 for the full 2,000-allocation sequence). The runs test showed no detectable first-order departure from independence (z = -0.09; P = 0.93), and lag-1 autocorrelation was 0.001 (P = 0.95; Table 1).

**Table 1:** Retrospective summary of the supplied demonstration log Table Credits: Kevin T. Malone.

Window or test	Value	Interpretation	Comment
First 1,000 allocations	502 vs 498	Near 1:1 allocation	Exact binomial P = 0.92
All 2,000 allocations	1008 vs 992	Near 1:1 allocation	Exact binomial P = 0.74
Runs test	z = -0.09	No obvious first-order serial dependence	P = 0.93
Lag-1 autocorrelation	r = 0.001	Essentially zero	P = 0.95
Observed time span	538 s	Short demonstration run	00:15:07 to 00:24:05

**Figure 1:**
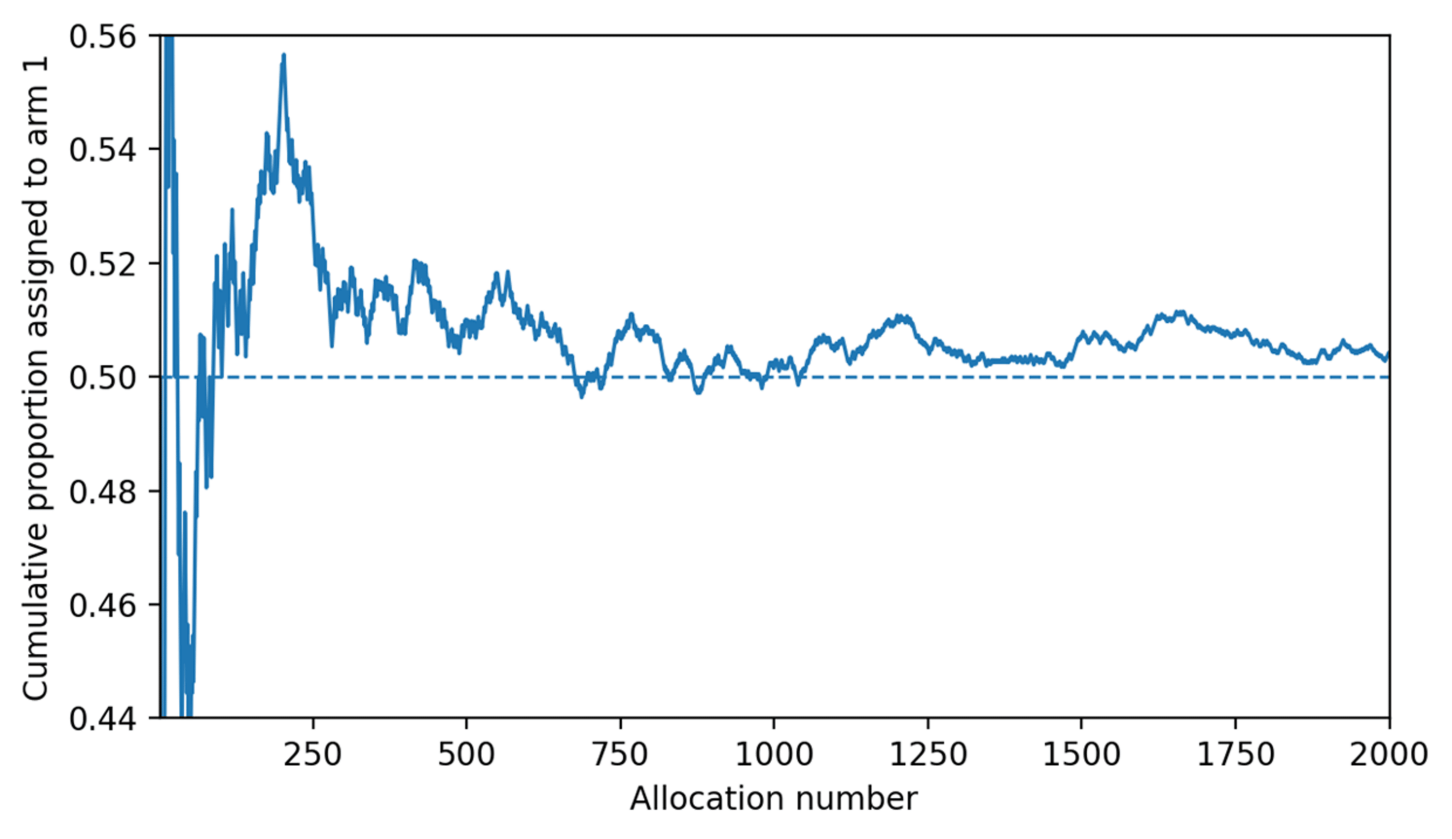
Cumulative proportion assigned across the 2,000-event demonstration log Credit: Kevin T. Malone, created using Python

Figure 1 shows the cumulative proportion assigned to arm 1 across the full sequence. The curve oscillates around 0.50 without sustained directional drift. Taken together, these findings are consistent with acceptable short-run behavior for a binary simple-randomization stream.

These findings do not, however, constitute proof of randomness, nor do they establish superiority over other generators. Comprehensive validation of a random number generator requires longer sequences, multiple seeds, and testing batteries designed specifically for empirical RNG assessment [14,15]. A short near-50:50 demonstration log is reassuring, but it is not definitive.

The version mismatch between the current source file and the supplied log also limits the strength of the demonstration. The manuscript would be stronger if the empirical log were regenerated directly from the version-controlled source analyzed in the paper, and both artifacts were archived together.

## Discussion

Relative to many ad hoc local spreadsheets and small desktop utilities, the Malone Randomizer offers several practical advantages. The full logic chain is short enough to inspect end-to-end, the assignment rule is explicit, the raw generator output is retained, and each event is recorded in a human-readable format. The addition of chained hashing further strengthens sequential traceability by linking each entry to the one before it. These features do not establish superior randomness, but they do improve transparency, explainability, and the ease of retrospective review. Because each hash incorporates the previous digest, later entries depend on earlier ones, and the log functions as a connected sequence rather than as isolated lines. Editing or deleting an earlier entry would be expected to disrupt the downstream chain, making later alteration easier to detect on review. This property is best described as tamper-evident or tamper-resistant rather than tamper-proof in the current local implementation.

If session seed custody were separated from the visible operating log and preserved independently, some forms of manipulation would become more difficult because local users could no longer easily reconstruct the full sequence from the beginning of the session. That design change could improve role separation and strengthen post hoc verification. Even so, seed concealment alone would not eliminate the risks of a local, user-resettable system.

Compared with unrestricted simple randomization, the Malone Randomizer is similar at the design level: each allocation is generated without blocking, stratification, or covariate adaptation. Its advantage does not lie in a different randomization theory, but rather in making the path from raw generator output to treatment assignment unusually explicit.

Compared with blocked and stratified randomization, the current program is less capable of controlling imbalance across sites or important prognostic covariates [4-6]. This distinction matters in many medical studies, particularly when sample sizes are modest and investigators need to balance not only in overall counts but also within clinically meaningful subgroups.

Compared with minimization and other covariate-adaptive approaches, the present prototype is intentionally simpler and easier to inspect, but it sacrifices the balancing performance that those methods can provide when covariates are clinically important [7-9].

Compared with dedicated randomization software for parallel-group trials, the current prototype is more transparent in source form but less capable operationally. Mature software packages commonly support participant identifiers, block schemes, stratification, output formats, and administrative controls that are absent from the present implementation [10].

Compared with spreadsheet-based workflows, the most defensible claim is one of inspectability rather than categorical statistical superiority. Earlier Excel implementations had documented weaknesses, whereas later versions showed substantial improvement in random number generation [12,13]. For that reason, it is not factually safe to claim that this prototype is simply more random than Excel. A stronger and more defensible claim is that the allocation pathway is easier to inspect line by line than in many ad hoc spreadsheet workflows. Earlier spreadsheet-based randomization reports likewise emphasized the importance of integrity checks and shadow verification [11].

Compared with central electronic data capture (EDC), interactive web response system (IWRS), or other server-based allocation systems, the current build is weaker in concealment, access control, governance, and immutable audit architecture (Table 2). That gap is clinically important because even a statistically acceptable allocation sequence can still be subverted if future assignments are locally visible or operationally guessable [2,3].

**Table 2:** Position of the current prototype relative to common randomization approaches Table Credits: Kevin T. Malone. Synthesis from references [2-13]. EDC: Electronic Data Capture; IWRS: Interactive Web Response System

Approach	Typical strength	Typical limitation	Relative position of this prototype
Simple randomization	Maximal design simplicity	May produce imbalance in small trials	Similar overall design, but with more explicit logging
Blocked or stratified randomization	Better count or subgroup balance	Can become predictable if concealment is weak	Less capable for balance, but easier to inspect
Minimization or covariate-adaptive allocation	Better covariate balance	Greater implementation and governance complexity	Simpler, but with less balancing power
Spreadsheet workflow	Widely available and familiar	Variable transparency and audit quality	Potentially more inspectable
Dedicated desktop randomization software	More features for trial operations	Often still local and not always transparent in source form	Less feature-rich, but more open to inspection
Central EDC/IWRS service	Strong concealment and governance	Higher development and maintenance burden	Currently inferior in operational readiness

This prototype improves some research workflows in five concrete ways. First, the code is brief enough that a clinician, informatician, or statistician can inspect the full logic chain without substantial reverse engineering. Second, it relies only on common Python standard-library components. Third, it exposes the raw 64-bit integer underlying each assignment rather than hiding the allocation decision inside a black-box function. Fourth, the current source code incorporates digest chaining to strengthen sequential traceability. Fifth, because each step is explicit rather than implicit, the program is readily open to independent code review. These features are methodological strengths only in the limited sense of improving transparency, explainability, traceability, and reviewability; they should not be interpreted as direct empirical evidence of improved clinical utility, superior sequence generation, stronger allocation concealment, cryptographic robustness, resistance to manipulation, readiness for regulated clinical-trial deployment, or broader methodological superiority.

These are meaningful advantages, particularly for educational use, pilot-study infrastructure, or other settings in which research teams need a simple and inspectable binary allocator. The important qualification, however, is that these are improvements in transparency, explainability, and traceability rather than in every dimension of clinical trial readiness. For clinicians, the main takeaway is not that this prototype provides a superior randomization algorithm, but that randomization tools used in clinical research should be understandable, auditable, and difficult to manipulate after the fact. Clinicians who enroll patients into trials are often asked to trust allocation systems that are operationally opaque. This work shows how a simple allocation event can be made more transparent: the raw generator output, the assignment rule, the timestamp, and the chained hash are all visible for later review. In small investigator-initiated studies, quality-improvement projects, simulation studies, or educational research, that level of inspectability may help clinicians understand how assignments were generated and may make retrospective review more credible. At the same time, clinicians should not interpret this prototype as a replacement for centralized randomization systems in concealment-sensitive or regulated clinical trials. The practical lesson is narrower but important: clinical randomization infrastructure should be judged not only by whether it produces balanced groups, but also by whether the allocation process can be explained, audited, and protected against avoidable manipulation. These limitations have practical consequences for real-world clinical research. Timestamp-derived seeding and parity-based assignment may be acceptable for demonstration, education, simulation research, or low-risk pilot workflows, but they would be inadequate for studies in which future assignments must remain concealed from enrolling clinicians. Similarly, the chained hash log improves tamper evidence, but it does not create an immutable audit record when the log remains local, user-resettable, and unanchored to external timestamping or write-once storage. The current prototype also lacks authentication, role separation, patient binding, central allocation control, blocking, stratification, and minimization. These omissions limit its applicability in multicenter, regulated, concealment-sensitive, or prognostically sensitive clinical trials. Accordingly, the system should be interpreted as an inspectable allocation prototype and research-informatics demonstration, not as a replacement for validated centralized randomization infrastructure.

Stated plainly, the Malone Randomizer is currently more compelling as an open, reviewable allocation prototype than as a finished randomization service for regulated interventional trials. Below is an illustrative example of a log entry that a reviewer might audit.

… 5. 2026-04-02 01:48:07 - Result: 2

 Raw Output: 14216707056671067707

 Computation: (14216707056671067707 % 2) = 1 -> 2

 Used Bit: 1

 Hash Chain: 8bc5415547bfb85da9ea47c6023d66d329b24539311a9dcda37b19ab4f4c8c53

6. 2026-04-02 01:48:07 - Result: 1

 Raw Output: 1394852543634368530

 Computation: (1394852543634368530 % 2) = 0 -> 1

 Used Bit: 0

 Hash Chain: f0ad29670e38cc195b5789d90e8a69dd293e4c802e699057e24482a014f658d5

7. 2026-04-02 01:48:08 - Result: 2

 Raw Output: 13280951112923668849

 Computation: (13280951112923668849 % 2) = 1 -> 2

 Used Bit: 1

 Hash Chain: 5eeaf68a17f1fdfdad0b7cbe41bff8da6638af9bf8c49ed5561bd69debbf02b5 …

For the above code for randomization, there are several pieces of information given. Let's take the first example "5". This is the entry number. It shows that this was the fifth allocation recorded in the current session. "2026-04-02 01:48:07" this is the timestamp. It records the exact date and time at which the allocation occurred. "Result: 2" this is the final treatment assignment shown to the user. In this system, the result is always either 1 or 2. "Raw Output: 14216707056671067707" this is the full integer produced by the random-number generator for this allocation event. It is the underlying number from which the final assignment was derived. "Computation: (14216707056671067707 % 2) = 1 -> 2" this line shows exactly how the assignment was made. The program divides the raw output by 2 and looks at the remainder. If the remainder is 0, the number is even and the assignment is Result 1. If the remainder is 1, the number is odd and the assignment is Result 2.

In this example, the remainder was 1, so the program assigned Result 2. "Used Bit: 1" this is the least significant bit of the raw output. It gives the same information as the remainder in simpler binary form. 0 means the number was even and maps to "Result 1". 1 means the number was odd and maps to "Result 2". "Hash Chain: 8bc541..." this is the chained SHA-256 digest for this entry. It is generated from the current event and the previous entry’s hash value, linking the records together in sequence. Its purpose is to make later alteration of the log more evident during review. Importantly, the log is more tamper-resistant because each entry is linked to the one before it. In the present system, the Hash Chain for a new entry is generated from the current randomization event together with the previous entry’s hash. As a result, the entries do not function as isolated lines, but as part of a connected sequence. If an earlier entry were edited or deleted, the downstream hash values would no longer match the expected chain, making the alteration easier to detect on retrospective review. If the original session seed were withheld from experimenters and preserved separately, some forms of manipulation would become more difficult because the experimenter could no longer easily reconstruct the full sequence from the beginning of the session. In that sense, separating seed custody from the visible operating log could strengthen traceability and make post hoc verification more credible. However, seed concealment alone would not make the system tamper-proof. In the current prototype, experimenters could still influence the process through local access, because the tool is locally hosted, the log can be cleared from within the interface, and both the output and assignment are visible at the time of use. Thus, even if the original seed were withheld, an experimenter could still delete records, restart sessions, or otherwise alter the operational record. These risks could be mitigated in a cloud-based architecture managed by an independent third party, with external timestamping, append-only audit storage, and seed access restricted to designated users. In the present manuscript, however, the program is intentionally presented in simplified local form for conceptual clarity and proof-of-concept review. The present prototype is notable because it prioritizes inspectability without abandoning explicit algorithmic structure. That design choice aligns with the broader informatics view that logs are not merely by-products, but data structures that support accountability, traceability, and retrospective reconstruction of events [18,19]. The current source file’s use of chained hashing is therefore not merely cosmetic. Rather, it points toward a more robust audit model, even if the present local implementation is not yet sufficient on its own [20,21]. In that sense, the novelty of the prototype does not lie in a new theorem of random allocation. Rather, it lies in a deliberately inspectable implementation that makes the randomization event itself reviewable as a clinical informatics artifact. The prototype has real strengths, but a high-level medical manuscript should also state clearly what remains unfinished before the system could credibly support concealment-sensitive clinical trial allocation. The prototype’s current limitations can be grouped into several domains. Generator validation: the current engine is xorshift-inspired, but it is not implemented in canonical xorshift* form, and no independent multi-seed validation against standard test batteries was supplied [14-17]. Bit-selection strategy: treatment assignment is determined entirely by parity, so only the least significant bit of each output controls allocation. In xorshift* analysis, that bit is a known weak point for scrambling [17]. Seed predictability: timestamp-derived seeding is reproducible but not cryptographically robust. If launch time is approximately known, the seed space may be narrower than desirable for concealment-sensitive work [2,3]. Operational concealment: the local desktop design exposes allocation generation to the same endpoint that may be used for enrollment. No authentication, role separation, patient binding, or central service is present [2,3]. Audit immutability: the log is stored locally, can be cleared from the interface, and is not anchored to an external timestamping or write-once mechanism [18-21]. Clinical design flexibility: the current build supports only simple binary allocation and does not implement blocks, stratification, or minimization [4-9]. Version control and evidentiary provenance: The demonstration log was intentionally presented in a simplified, reader-facing format so that clinicians and reviewers could understand the meaning of each allocation field. However, this means that the displayed example should not be treated as the canonical audit record generated by the current source code. For formal validation, the exact raw log, source-code version, hash-chain format, and any reformatted publication excerpts would need to be preserved together so that the original evidence can be distinguished from explanatory displays.

## Conclusions

The Malone Randomizer is a credible and interesting prototype because it treats randomization as a reviewable event rather than as a hidden software output. The code is short, the mapping from integer output to treatment assignment is explicit, and the current implementation moves toward tamper-evident chained logging. The evidence supports a conservative conclusion: this prototype is a low-dependency binary allocation tool whose principal novelty lies in transparency. The current materials do not support stronger claims of categorical statistical superiority over modern spreadsheet-based or enterprise systems, nor do they support the phrase “proof of randomization.” With stronger seeding, improved output mapping, richer trial-design options, more durable audit architecture, and validation from version-matched artifacts, the prototype could mature into a useful platform for pilot studies, small trials, and research-informatics deployment.

## Appendices

The Appendix summarizes the software architecture and logged fields in tabular form. The full source code and test data analyzed in this manuscript are publicly available in the Malone Randomizer GitHub repository: https://github.com/malonekt/research-randomizer.

**Table 3:** Summary of core program components Table Credits: Kevin T. Malone.

Component	Function	Relevance
seed_from_time()	Derives a 64-bit seed from the current timestamp	Lightweight, reproducible, but not cryptographically strong
next()	Advances the xorshift-inspired state and returns a 64-bit value	Produces the integer used for assignment
generate_random()	Converts output parity to arm 1 or 2 and writes the log entry	Core allocation step
hash_chain()	Computes SHA-256 using current value and prior hash	Supports tamper-evident chaining
clear_log()	Resets the local log, counter, and chain state	Limits immutability
GUI layer	Displays assignment and most recent log entry	Minimal local desktop interface

**Table 4:** Fields recorded in each allocation log entry Table Credits: Kevin T. Malone.

Field	Meaning	Audit purpose
Entry number	Sequential allocation count within session	Event ordering
Timestamp	Date/time of allocation	Temporal traceability
Result	Assigned arm (1 or 2)	Final visible allocation
Raw output	Underlying PRNG integer	Inspectability
Computation	Explicit % 2 mapping to result	Reproducible assignment logic
Used bit	Least significant bit / parity	Shows the deciding bit
Hash chain	Chained SHA-256 digest	Tamper-evident linkage
